# Effect of root canal sealer and artificial accelerated aging on ﬁbreglass post bond strength to intraradicular dentin

**DOI:** 10.4317/jced.51465

**Published:** 2014-10-01

**Authors:** Fernanda-Ribeiro Santana, Carlos-José Soares, Josemar-Martins Ferreira, Andréa-Dolores-Correia- Miranda Valdivi, João-Batista-de Souza, Carlos Estrela

**Affiliations:** 1 DDS, MSc, PhD. Department of Stomatologic Sciences, School of Dentistry, Federal University of Goiás, GO, Brazil; 2 DDS, MSc, PhD. Operative Dentistry and Dental Materials Department, School of Dentistry, Federal University of Uberlândia, Uberlândia, MG, Brazil; 3 DDS, MSc, PhD, Post-graduate student. School of Dentistry, Federal University of Uberlândia, MG, Brazil; 4 DDS, MSc, Post-graduate student. School of Dentistry, Federal University of Uberlândia, MG, Brazil; 5 DDS, MSc, PhD. Department of Prevention and Oral Rehabilitation, School of Dentistry, Federal University of Goiás, GO, Brazil

## Abstract

Objectives: To evaluate the effect of root canal sealers (RCS) and specimen aging on the bond strength of fibre posts to bovine intraradicular dentin. Material and Methods: 80 teeth were used according the groups - Sealapextm, Sealer 26®, AH Plus® and specimens aging - test with no aging and with aging. The canals prepared were ﬁlled using one of each RCS. The posts were cemented. Roots were cross-sectioned to obtain two slices of each third. Samples were submitted to push-out test. Failure mode was evaluated under a confocal microscope. The data were analysed by ANOVA, Tukey’s, and Dunnet tests (α = 0.05). Results: No signiﬁcant difference was detected among RCS. Aged control presented higher bond strength than immediate control. The aging did not result significant difference. Adhesive cement-dentin failure was prevalent in all groups. Conclusions: RCS interfered negatively with bonding of fibreglass posts cemented with self-adhesive resin cement to intraradicular dentin.

** Key words:**Fibreglass post, bond strength, root dentin, endodontic sealer, aging.

## Introduction

Root canal filling is an important step in the last phase of endodontic treatment. This procedure is achieved with the association of a solid filling material, such as gutta-percha, and root canal sealer (RCS). One of the key roles of the RCS is to aggregate the root filling material and maintain it as compact mass with no gaps, which adheres to the root canal walls and provides a monoblock configuration that seals hermetically the canal space (1). This adhesion process involves mechanical forces that yield the intertwining of the material with dentin structures and may result in a greater sealing ability, thus reducing the risk of root canal microleakage and maintaining a cohesive filling mass (2).

As endodontically treated teeth with loss of part of the coronal structure generally require a post for restoration of tooth function (2). The compatibility among different materials used in root canal treatment and in luting fibre posts to root dentin is an important aspect to be considered for a successful restoration of root treated teeth. Depending on its composition, the RCS might interfere with the bonding of a post to root dentin (3-7).

RCS based on epoxy resin are employed because of their good physicochemical properties (5) and good adhesion (8,9). Calcium hydroxide-based sealers have the capacity to stimulate the formation of mineralized tissue (10). However, there have been only a few studies regarding the influence of these sealers on bond strength of fibre posts cemented with self-adhesive resin cements.

The self-adhesive resin cements were introduced into the dental market in 2002 with the advantage that no pretreatment of the tooth surface is required for the cementation of fibreglass posts (6) and with a bonding mechanism based on micromechanical retention and chemical adhesion (11). After post preparation, a residual filling material may become displaced into the root canal, which may influence negatively the bond strength mainly in deeper area (12). The residual debris tend to be more inappropriate for the self-adhesive system, because there is no etching and rinsing before resin cement application (12).

This study analysed the effects of the endodontic sealer and the specimen artificial accelerated aging on bond strength of fibre posts cemented with a self-adhesive resin cement to root canals of bovine incisors to test whet-her these factors had any effect on ﬁbre post bond strength to root dentin, according to the different regions of the post space (cervical, middle, apical). The null hypothesis tested was that the root canal sealers and the specimen artificial accelerated aging have no inﬂuence on ﬁbreglass post regional bond strength to intraradicular dentin.

## Material and Methods

Eighty freshly extracted bovine incisors with roots that were anatomically similar in size, shape and had a canal less than 1 mm in diameter and fully developed apices were selected from 300 teeth and stored in distilled water. Each tooth was decoronated using a double-faced diamond disc (KG Soresen, São Paulo, SP, Brazil) operated perpendicularly to its longitudinal axis to produce roots 15 mm long. Roots were randomly divided into eight groups (n=10), two controls (no root canal filling) and six experimental groups resulting from the interaction between two study factors: endodontic sealer – Calcium hydroxide-based sealer, Sealapex, Kerr Corporation, Orange, USA (SX); Calcium hydroxide-based sealer, Sealer 26, Dentsply Maillefer, Petrópolis, RJ, Brazil (S26); Resin-based sealer, AH Plus, Dentsply DeTrey GmbH, Konstanz, Germany (AH); and specimen aging - test with no aging (immediate) and test performed after artificial accelerated aging with 2 months of water storage at 37°C (aged).

- Endodontic procedures

Root canals were prepared to 1 mm short of the apex using a crown-down technique with sizes # 1,2 Gates Glidden burs (Dentsply Maillefer) (WL 10 mm) and K3 nickel-titanium rotary instruments (SybronEndo, Optimum, São Paulo, SP, Brazil) in the following sequence: # 25 .10 taper (WL 10 mm); # 15-25 .02 taper (WL 14 mm); # 25 .04 taper (WL 14 mm); # 25 .06 taper (WL 14 mm); # 30-45 .02 taper (WL 14 mm). During root canal preparation (RCP), at each change of instrument, root canals were irrigated with 2 mL of 1% sodium hypochlorite (NaOCl) (Fitofarma, Goiânia, GO, Brazil). In all groups, 3 mL of 17% EDTA (Biodinâmica Química e Farmacêutica Ltda., Ibiporã, PR, Brazil) was used for 5 minutes to remove the smear layer. The final irrigation was performed with 5 mL of 1% NaOCl (Fitofarma).

In control groups (immediate and aged), after RCP, the root canals were not filled. In the six experimental groups, roots canals were dried with absorbent paper points and ﬁlled with gutta-percha (Dentsply Maillefer) and the speciﬁc RCS prepared and used according to manufacturers’ instructions, using the lateral compaction technique. After root canal ﬁlling, a coronal seal was created with glass ionomer cement (Vidrion R; SSWhite, Rio de Janeiro, RJ, Brazil) and samples were stored in distilled water at 37°C for 24 hours. After 24 hours, the ﬁlling material was removed with Gates-Glidden burs to a depth of 10 mm with 5 mm of apical seal maintained.

- Post space preparation

In all groups post space was prepared using sizes # 3-5 Largo burs (Dentsply Maillefer) (WL 10 mm), which corresponded to 1.5 mm parallel-sided, serrated fibre post (Reforpost # 3; Angelus, Londrina, PR, Brazil). Root canals were irrigated, at each change of burs and at the end of the preparation, with 2 mL of 1% NaOCl and dried with absorbent paper points. All roots were covered externally with utility wax to avoid lateral activation by light source.

- Post luting procedure

Fibreglass posts were cleaned with 70% alcohol, then in a single application using a Microbrush, and after drying, a silane agent was applied for 1 min (Silano; Angelus). The self-adhesive resin cement (RelyX U100; 3M-ESPE, St. Paul, MN, USA) was prepared according to the manufacturer’s instructions, introduced into the canal using a K-File and placed on the post. The post was seated to full depth by ﬁnger pressure. Excess cement was removed after 1 min. After 5 min, the resin cement was light-cured using a 1200 mW/cm2 (Radii-Cal; SDI, Bayswater, Australia) source for 40 seconds each on the cervical face of the root, in the direction of the long axis of the root, and obliquely to the buccal and lingual surfaces, for a total of 120 seconds. The coronal limit of the interface between the post-cement-dentin was then sealed using composite resin, and the roots were stored in distilled water at 37°C for 24 hours. The samples were stored for 24 hours until the resin cement had completely sett, because it is a dual cement, which after activation with curing needs a time to promote complete chemical setting.

- Specimen preparation for push-out test

After 24 hours, each root was serially sectioned perpendicular to its long axis using a double-faced diamond disc (102.0 mm Diameter x 0.3 mm Thickness x 12.7 mm Arbor, Extec, Enﬁeld, CT, USA) and a precision saw (Isomet 1000, Buehler, Lake Bluff, IL, USA) at low speed under water cooling to obtain two slices measuring 1 mm thick from cervical, middle and apical post thirds, a total of six slices per root.

- Push-out test

In immediate groups, slices were submitted immediately (with no aging) to a push-out test, while in aged groups, slices were stored in distilled water at 37°C for 2 months (artificial accelerated aging) prior to testing. The push-out test was performed in a testing machine (EMIC DL 2000, São José dos Pinhais, PR, Brazil) by applying a compressive load at 0.5 mm/min from the apical to coronal direction until failure. The bond strength was calculated in MPa by dividing the load at failure (N) by the area of the bonded interface. The area of the bonded interface was calculated as follows: A = 2πrh, where A is the area of the bonded interface, π = 3.14, r is the radius of the post segment (mm) and h is the thickness of the post segment (mm) (12,13).

- Statistical analysis

SAS software (Release 9.2, SAS Institute Inc., Cary, NC, USA) was used for statistical analysis. The Shapiro-Wilk test was used to test normality. The effects of endodontic sealers and specimen artificial accelerated aging were analysed using one-way analysis of variance in a split-plot arrangement, with the main plot for the endodontic sealer or the aging, and the subplot for post third (cervical, middle and apical). The Tukey test was used for multiple comparisons (α = 0.05). Comparisons with control groups (immediate and aged) were made using the Dunnet test (α = 0.05).

- Confocal microscopy

To determine failure mode, all fractured specimens were only air dried and analyzed under a confocal laser scanning microscope (Carl Zeiss Laser Scanning Systems, LSM510, META, Oberkochen, Germany). Images were analyzed using the Zeiss LSM Image Browser (META, Germany). The failure mode was classiﬁed into six types: (I) adhesive between the post and resin cement; (II) adhesive between resin cement and intraradicular dentin; (III) cohesive in cement; (IV) cohesive in dentin; (V) cohesive in post and (VI) mixed, among post, resin cement and intraradicular dentin (14,15).

## Results

Mean push-out bond strengths and standard deviations of immediate and aged groups are shown in  Table 1 and  Table 2, respectively. One-way analysis of variance (endodontic sealer) with subplot (post third) of immediate and aged groups showed a significant difference only for post third (*P* < .001). Significant reductions on bond strength values from the cervical to apical third, regardless of the endodontic sealer, were found. Comparisons of experimental groups with immediate and aged control groups are presented in  Table 1 and  Table 2, respectively. The Dunnet test showed that the use of an endodontic sealer resulted on lower bond strength values than control groups (no root filling), with statistically significant difference in middle (*P* = .0294) and apical thirds (*P* = .0207) of immediate groups ( Table 1), and in cervical (*P* = <.0001), middle (*P* = <.0001) and apical (*P* = <.0001) thirds of aged groups ( Table 2).

**Table 1:**
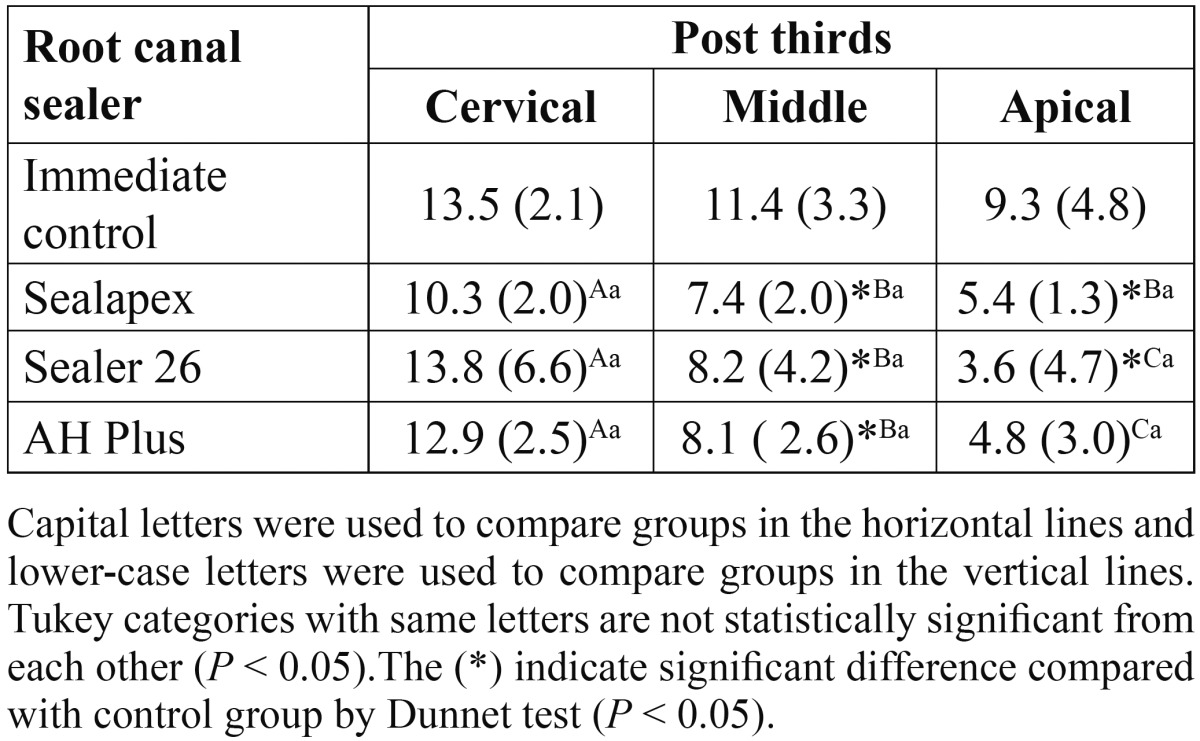
Bond strength means in MPa (standard deviation) of immediate groups (no aging) and statistical categories deﬁned by Tukey’s test and Dunnet test (n = 10).

**Table 2:**
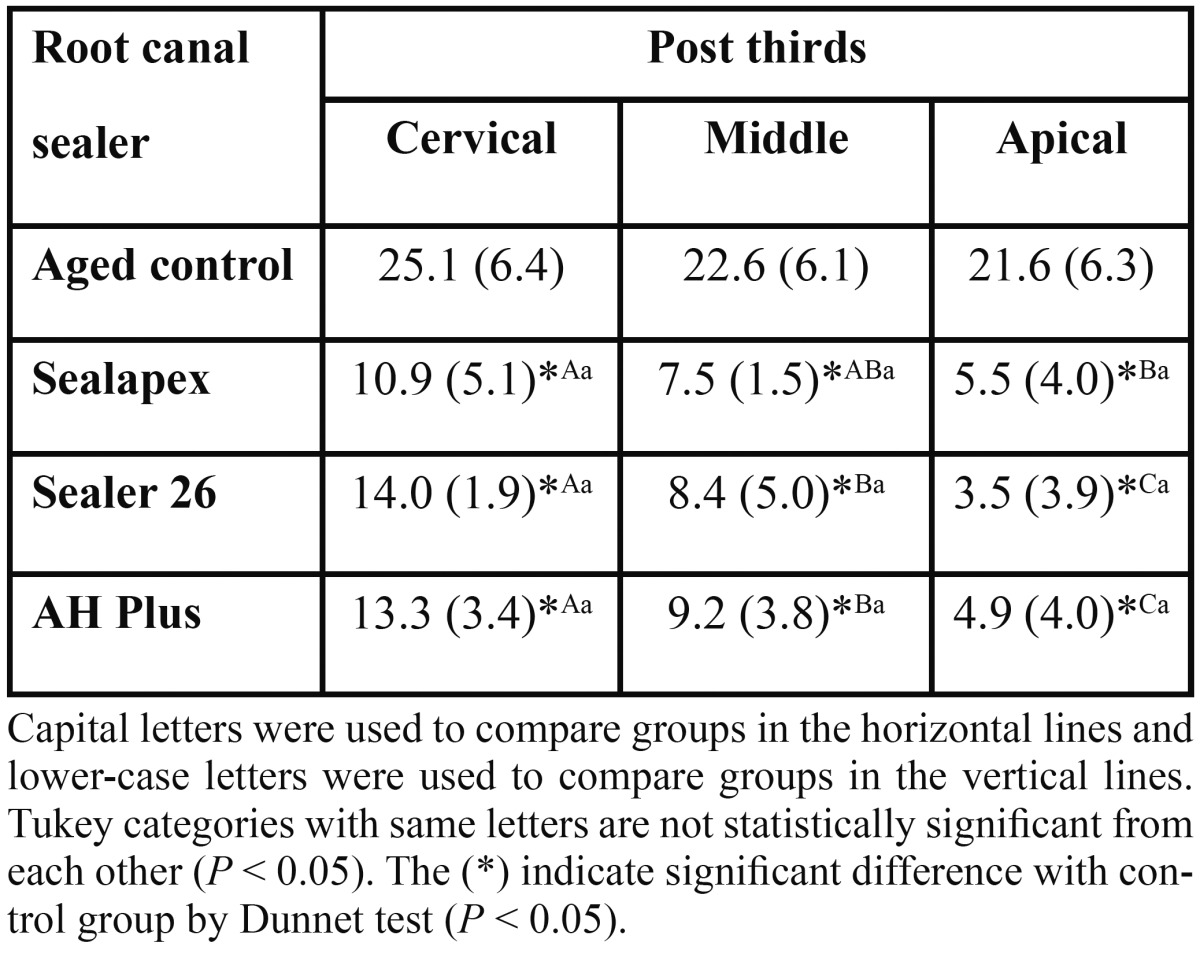
Bond strength means in MPa (standard deviation) of aged groups and statistical categories deﬁned by Tukey’s test and Dunnet test (n = 10).

**Figure 1:**
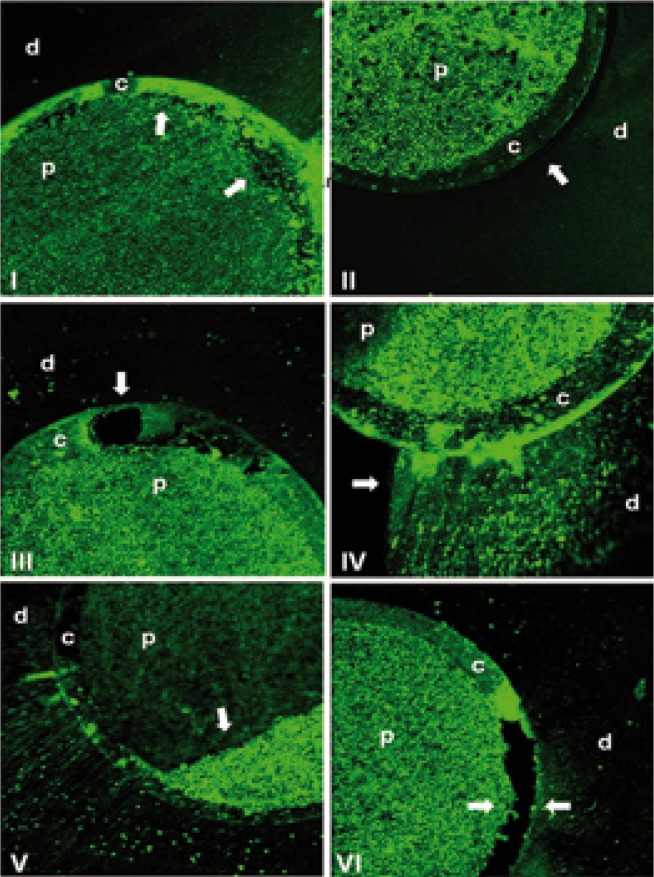
d – dentin; c – resin cement; p – fibreglass post. Microscopic images of failure mode after push-out test (magnification 10X): (I) adhesive between the post and resin cement; (II) adhesive between resin cement and intraradicular dentin; (III) cohesive in cement; (IV) cohesive in dentin; (V) cohesive in post; (VI) mixed among post, resin cement and intraradicular dentin. Arrows point to failure region.

Mean push-out bond strengths and standard deviations as a result of specimen aging and post third of all groups are in  Table 3. One-way analysis of variance (artificial aging) with subplot (post third) for each RCS (SX, S26, AH) showed significance only for post third (*P* = <.001). In control, there was significance for artificial aging (*P* = <.001) and post third (*P* = .005). Tukey’s test revealed a significant reduction on bond strength values from cervical to apical third, regardless of the artificial aging. Aged control group presented higher bond strength than immediate control. Failure distribution is shown in  Table 4 and figure 1. Adhesive cement-dentin failure was prevalent in all groups.

**Table 3:**
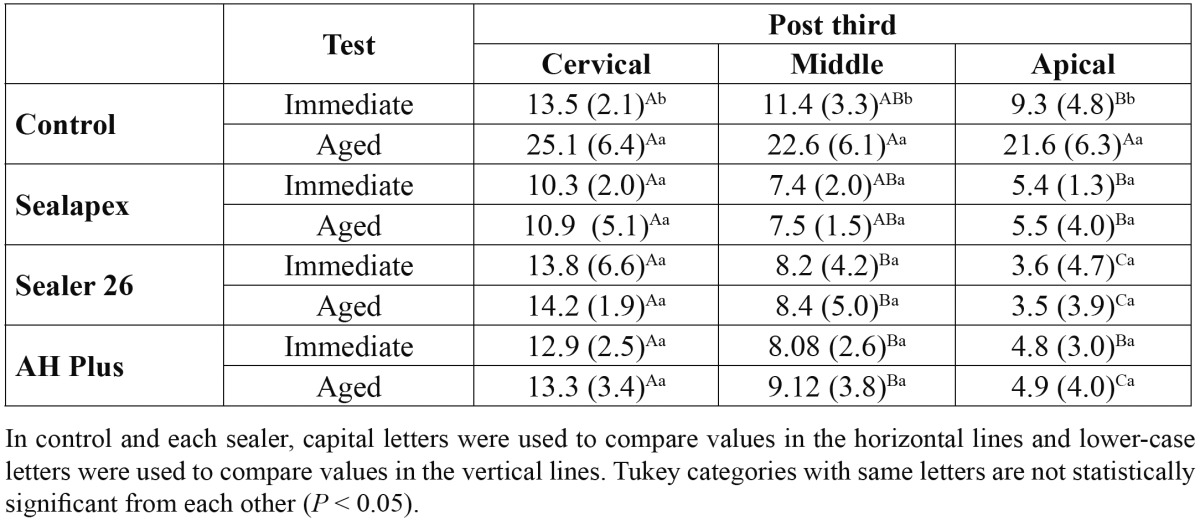
Bond strength means in MPa (standard deviation) as a result of artificial accelerated aging and post third, and statistical categories deﬁned by Tukey’s test for control (no root filling) and each root canal sealer (n = 10).

**Table 4:**
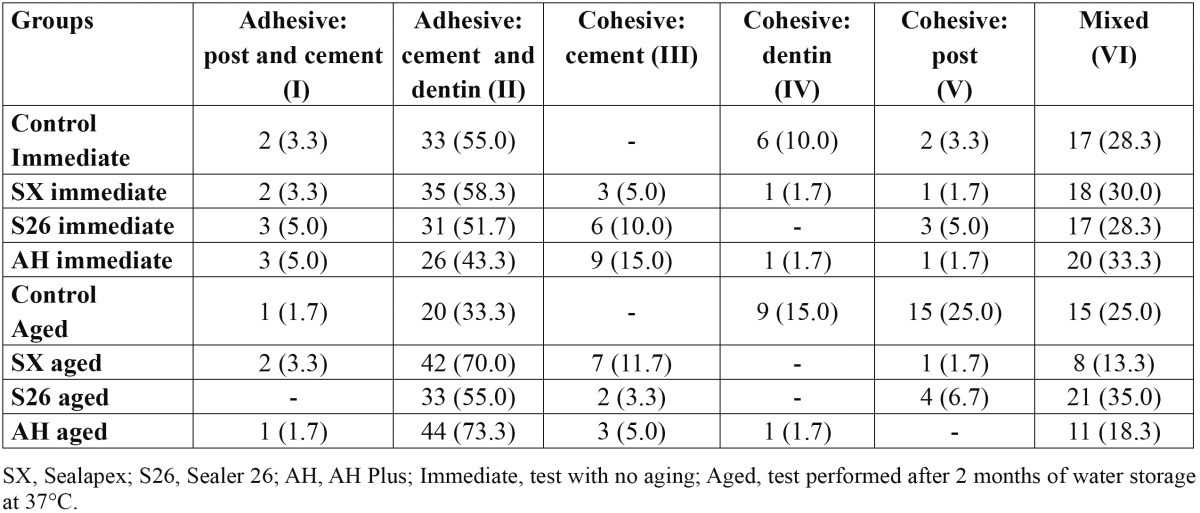
Failure mode (%) for groups.

## Discussion

The null hypotheses tested on this study were rejected. RCS and the artificial accelerated aging of samples affected the bond strength of ﬁbre posts to root dentin. Moreover, bonding varied according the different thirds of the post space.

Various mechanical methods have been used to measure in vitro the bond strength of fibreglass post to intraradicular dentin, as microtensile bond strength and push-out tests (16). The push-out method was shown to have fewer premature specimen failures, a lower data distribution variability (13,16) and a more homogenous stress distribution (16) compared to microtensile method during the bond strength evaluation of fibreglass posts to intraradicular dentin. The push-out laboratory test would seem to be a more appropriate methodology for the evaluation of fibreglass posts bonded to intraradicular dentin (16).

When RCS are introduced into the root canal and obturation forces are applied, it is likely that sealers penetrate into the dentinal tubules (4). Endodontic sealer penetration increases the interface between material and dentinal walls, what may improve the mechanical retention of the material by interlocking of the sealer plug inside the tubules and potentially reduces leakage (17). The ability of the endodontic sealer to penetrate dentinal tubules consistently and effectively is one of many factors inﬂuencing the choice of material for root canal ﬁlling (17). The use of EDTA, that is able to act on tooth mineral matrix and promote removal of the smear layer formed during RCP, allows a better penetration of endodontic sealers into the dentinal tubules, increasing the contact surface of the filling material with dentin (9).

The removal of the sealer impregnated in root dentin during post-space preparation is a critical step in achieving optimum post retention when resin cement is used (2). However, previous studies revealed, by scanning electron microscopy (SEM) analysis, the difficulty in obtaining a dentin surface cleaned and suitable for resin adhesion in endodontically treated teeth after the post space preparation (5,18). In the present study there was a significant decrease of post retention for all endodontic sealers compared to control groups, in which no endodontic sealer was used, which suggests that post-space preparation may not have removed all sealer-impregnated dentin, and not ensuring a “freshened” surface to which resin cement could bond (2).

The action of drills used to remove the root ﬁlling material to create a post space produces a new smear layer rich in endodontic sealer and gutta-percha remnants plasticized by the friction heat of the drill (5,18). Independently from the type of endodontic sealer, this smear layer can act as physiochemical barrier against any kind of adhesive material intended to bond to intraradicular dentin (7). Moreover, in the present study, root canals were irrigated after post preparation only with 1% NaOCl that acts selectively on the removal of organic particles (19,20) and cannot dissolve inorganic particles (21). Therefore, it does not effectively remove the smear layer formed on root canal walls after mechanical post preparation (9), which may reduce the penetration and chemical action of the self-adhesive resin cement (RelyX U100, 3M-ESPE).

Although this cement contains multifunctional hydrophilic monomers with phosphoric acid groups, it shows a low demineralization effect (11) with a superﬁcial morphological interaction. In agreement, Bitter et al. (15) described that with RelyX Unicem (3M-ESPE), which is chemically identical to the RelyX U100 differing only in the application procedure, the smear layer did not dissolve consistently at the cement-dentin interface and hybridization of dentin was only detected sporadically.

Additionally, an intense chemical interaction of RelyX Unicem with hydroxyapatite has been documented (11). Chemical interactions may be effective inside the root canal, and indicate that this interaction might be more crucial for root dentin bonding than the ability of the same material to hybridize dentin (15); however, an effective removal of the smear layer is necessary to allow that RelyX U100 becomes adapted to dentin, with good interfacial continuity, which can optimize physical interactions such as van der Waals forces, hydrogen bridges and charge transfer, enhancing micromechanical retention and chemical bonding (22). Probably, the post space preparation technique used in the present study did not allow a perfect removal of smear layer and endodontic sealer/gutta-percha remnants of dentin walls that may have affected the resin cement adhesion.

In agreement with this study, a previous report revealed that fibre post bond strength in the group without a root canal filling was higher compared to groups that used gutta-percha/AH Plus, gutta-percha/Guttaflow and preexisting root canal filling (7). It might be observed that RCS (AH Plus, Endofill and Sealapex) showed significant negative effects on fibreglass post bond strength (5). In disagreement, it was reported that AH Plus and Sealer 26 did not interfere with the adhesive bond of fibreglass posts compared to a control group that used only gutta-percha points (6). Menezes *et al.* (3) also did not find a significant difference between control group (unfilled) and groups filled with Sealer 26.

Bond strengths decreased significantly from cervical to apical post thirds, in agreement with findings from previous reports (3,4,13,23). Factors possibly associated with this decrease are the difficulty of access to apical level of post space (4); the difficulty in eliminating the smear layer and endodontic sealer/gutta-percha remnants covering dentin walls in this third (18); the nonuniform adaptation of the bonding material to the deepest portion of the post space (13) associated with possible limitations of cement ﬂow (4,23), as the cement used in the present study has a high viscosity (22); the drop in light intensity with increasing distance affecting the degree of conversion of resin cement (24).

In addition, the C-factor, deﬁned as the ratio of bonded to unbonded surface areas of cavities, in a root canal is highly unfavorable and contributes to maximizing the polymerization stress of resin based materials along the root canal walls (13,25). Morris *et al.* (26) estimated that C-factors in root canals can range from 20 to 100, depending on the diameter and length of the root canal.

Although ﬁber posts luted in root canals are not directly exposed to oral ﬂuids, water storage is considered as in vitro accelerated aging test for bonded interfaces (27,28). Previous studies reported that the exposure of specimens to water for 1 month (27) and 60 days (28) significantly reduced fiber post-resin (27) and fiber post-dentin (28) bond strengths due to formation of microcraks in polymer networks as a consequence of water sorption resulting in gaps at the post-adhesive interface (27) and hydrolytic degradation of collagen fibers (28). In the present study, water artificial aging for two months significantly increased post retention in control group (no root canal filling), but did not interfere with bond strength in experimental groups. Probably, the aqueous challenge proposed in this study was not enough to negatively affect the resin cement RelyX U100 network and, as it is a self-etch self-adhesive system, collagen fibers could be completely covered by the resin phase and are inaccessible to water needed to effect collagenolysis (28).

Increases in interfacial strength may be related to enhanced bonding ability or setting during water storage, stress relaxation by hygroscopic expansion as a consequence of water sorption during storage or hygroscopic expansion of luting materials (29). The hygroscopic expansion of the resin cement, in particular, could have contributed to a greater adaptation of the cement to dentin substrate in control group. A major contribution to retentive strength in push-out test is expected to occur as a consequence of the interfacial sliding friction (29) resulting from application of a compressive force. Thus, the higher 2-months interfacial strength achieved in control group may have been caused by the increase in interfacial friction, due to the greater adaptation of the cement to dentin consequent to hygroscopic expansion (29).

However, in experimental groups, surface debris and RCS/gutta-percha remnants on canal walls created a physiochemical barrier that may have affected the resin cement adhesion, as previous described, and probably also avoided this greater adaptation to dentin and micromechanical retention, despite the hygroscopic expansion. It is important to emphasize that the applied method is a simpliﬁed model of accelerated aging which has been commonly performed for challenging resin-dentin adhesion (27,28).

In the present study, confocal microscopy was used and appears to be a noteworthy alternative for failure modes analysis, since it is less time consuming and does not require any preparation of the specimens (14). The predominance of adhesive cement-dentin failures observed in all groups ( Table 4) may be attributed to the difficulty in eliminating the smear layer and endodontic sealer/gutta-percha remnants covering dentin walls (5,18,22), the reduced ability of dentin demineralization and hybridization of the self-adhesive cement RelyX U100 (15) and the highly unfavourable C-factor which maximizes polymerization contraction stresses and leads to debonding of resin from the canal wall (13,25,26).

One of the imitations of this study was that the specimens were not submitted to thermal and mechanical cycling to simulate intra-oral conditions more precisely. Bovine teeth are easier to collect and tooth age can be standardized (3,12). Moreover, studies have demonstrated similar properties between human and bovine teeth (30). Further studies need to be conducted to analyse the effects of the use of EDTA, after post space preparation, on the bond strength to intraradicular dentin in each third of post space.

## Conclusions

RCS (Sealapex, Sealer 26 and AH Plus) interfered negatively with bonding of fibreglass posts luted with self-adhesive resin cement to intraradicular dentin irrespective of post third. There was a predominance of adhesive failures between resin cement and dentin in all groups. Water artificial aging for 2 months increased post retention in control group (no root filling) and did not interfere with bonding in groups in which was used a endodontic sealer.
